# Irreducible Anteromedial Dislocation of Radial Head with Biceps Tendon Interposition

**DOI:** 10.1155/2016/5812353

**Published:** 2016-01-26

**Authors:** Vicente J. Climent-Peris, Josette Sirera-Vercher, M. Dolores Sanz-Amaro

**Affiliations:** Department of Orthopaedic Surgery, Hospital Lluís Alcanyís, Xàtiva, Valencia, Spain

## Abstract

The case presents an isolated irreducible anteromedial dislocation of radial head due to biceps tendon interposition on a 14-year-old female patient. After an unsuccessful closed reduction, a lateral approach of the left elbow was carried out through Kocher's interval. Given that no pathology was found on the radiohumeral joint, the approach was extended distally. This revealed that the biceps tendon was displaced laterally around the radial neck, preventing the reduction. Once the tendon was taken back to its anatomical position, the radial head reduction was performed successfully. The patient achieved a complete functional recovery. Possible injury mechanisms are discussed, as well as the importance of identifying such a rare injury.

## 1. Introduction

The isolated dislocation of the radial head is an uncommon injury; it usually presents itself as part of the Monteggia fracture. The anterolateral dislocation is more commonly associated with annular ligament injuries, but the anteromedial dislocation is rare.

A case of an irreducible anteromedial dislocation of the radial head due to biceps tendon interposition on a female aged 14 years is herein presented.

## 2. Clinical Case

14-year-old female patient who was standing on both hands on the ground with both of her elbows extended (i.e., doing a handstand) suffered acute pain on her left elbow, failed to stand, and fell on the ground. After the accident, the patient was taken to ER. During an initial examination, it was observed that her elbow was in a semiextended and pronated position. Furthermore, she suffered severe pain when trying any movement. No important deformity was observed and the patient did not show any signs of neurovascular compromise.

**Figure 1:**
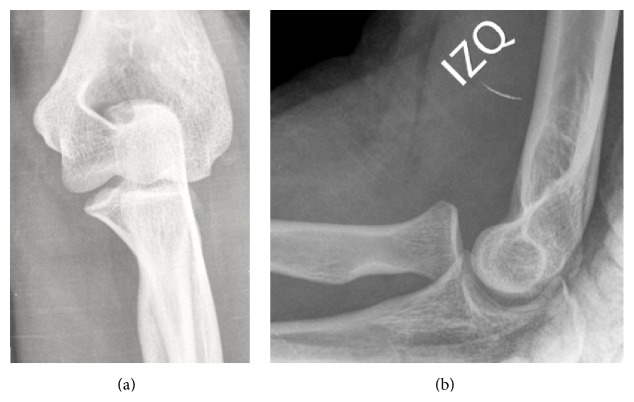
Left elbow anteroposterior (a) and lateral (b) X-ray where the medial and anterior dislocation of the radial head can be observed. No pathology is observed on the ulnohumeral joint.

Her left elbow was subjected to a radiological study, which included an anteroposterior and lateral X-ray (Figure 1) and a CT scan, which did not provide any further information.

Having made the diagnosis of isolated anteromedial dislocation of the radial head, an examination and reduction under general anesthesia were suggested. A closed reduction under radioscopic control was attempted and failed; therefore it was decided to perform an open reduction.

**Figure 2:**
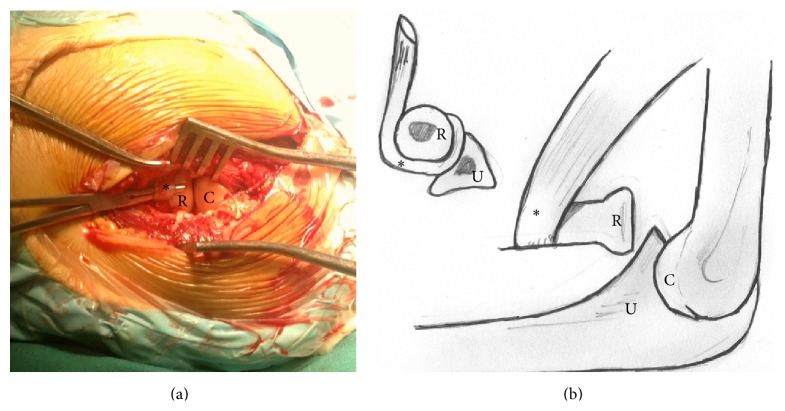
Intraoperative view (a) and injury diagram (b). The displaced biceps tendon prevents reduction of the radial head, keeping it in anteromedial position. R: radial head, C: humeral condyle, U: ulna, and *∗*: biceps tendon.

A lateral approach of the left elbow was carried out through Kocher's interval. A minimal osteochondral fracture of the radial head was observed, but no other peculiarities were noted on the joint. The dissection was extended distally, which allowed seeing the biceps tendon displaced laterally around the radial neck, therefore preventing the reduction (Figure 2). Once the tendon was reduced through the radius-humeral joint to its medial position, the radial head reduction was performed successfully. The annular ligament was repaired.

The joint was immobilized with a plaster cast at a position of 90 degrees of intermediate flexion and pronation-supination for two weeks, after which the patient began a progressive rehabilitation.

## 3. Results

Three months after surgery, the patient did not show any symptoms and had recovered full joint mobility. One year later the girl was asymptomatic with any pain or instability.

## 4. Discussion

The isolated dislocation of the radial head is a rare injury. Misdiagnosis, incorrect reduction, and relapse are frequent; they delay surgical intervention and therefore cause detrimental functional results [1–4].

The inability to carry out the reduction due to biceps tendon interposition is a rare situation, and some cases have been published [3–7].

As described by Sasaki et al. [4], we believe that the injury may be due to a fall in a hyperextended and supination position, which causes the anterolateral dislocation of the radial head. This is followed by hyperpronation which displaces the head medially, and the subsequent flexion of the elbow entraps the biceps tendon around the neck. This blocks the head in a medial position and prevents the closed reduction. On their studies on cadavers, Upasani et al. [7] described a mechanism consisting of hyperextension and forced valgus with elbow dislocation, entrapping the biceps tendon when reducing the dislocation.

In the cases published, the intraoperative management included the reposition of the biceps tendon to its anatomical position through the radiocapitellar joint in 3 cases [5–7], as in the case herein shown. In two cases, a tenotomy and subsequent reinsertion into the bicipital tuberosity were necessary due to a late diagnosis [3, 4].

The interposition of the annular ligament has also been described as the cause of the irreducible anterior dislocation of the radial head. The ligament suffers no injury and is interposed between the dislocated radial head and the capitellum [8].

These injuries should not be mistaken with a congenital radial head dislocation. This does not imply any preliminary trauma; it is often bilateral and anterolateral [3].

To conclude, when treating a case of isolated anteromedial dislocation of the radial head, we must take into account that there may be a lateral transposition of the biceps tendon which prevents the closed reduction. We must check thoroughly if we have achieved a correct and complete closed reduction. If an open reduction is intended, the exposition should be extended distally to the radial neck.

An accurate diagnosis of the injury leads to a simple resolution and to a complete functional recovery.
